# Emphysematous Cystitis Occurred in the Case Treated with Steroid for Autoimmune Hepatitis

**DOI:** 10.1155/2013/821780

**Published:** 2013-07-10

**Authors:** Tateki Yoshino, Shinya Ohara, Hiroyuki Moriyama

**Affiliations:** ^1^Department of Urology, JA Onomichi General Hospital, 1-10-23 Hirahara, Onomichi City, Hiroshima 722-0018, Japan; ^2^Department of Urology, Integrated Health Sciences, Institute of Biochemical & Health Sciences, Hiroshima University, Hiroshima 734-8551, Japan

## Abstract

Emphysematous cystitis is a rare clinically entity, more commonly seen in diabetic, immunocompromised patients, which was characterized by air within the bladder wall and lumen. A 83-year-old woman was introduced to our department with fever elevation and abnormal findings of computed tomography (CT). She took orally prednisolone for autoimmune hepatitis. Pelvic CT revealed diffuse air throughout the bladder wall. Urinalysis showed combined hematuria and pyuria. *Escherichia coli* was detected in blood culture. Abnormal findings of complete blood count and laboratory examination included an elevated WBC count (12,200/*μ*L), C-reactive protein (11.7 mg/dL), and creatinine (1.07 mg/dL). Cystoscopy confirmed diffuse submucosal emphysema throughout. On the basis of diagnosis with emphysematous cystitis, she was treated with antibiotics based on the results of blood culture and indwelling Foley catheter. After treatment, the improvement of inflammatory findings and submucosal emphysema on cystoscopy and CT were achieved.

## 1. Introduction

Air within the urinary tract can occur because of instrumentation, fistula, tissue infarction with necrosis, or infection. Gas-forming infections of the urinary tract, such as emphysematous cystitis and emphysematous pyelonephritis, are uncommon but potentially life threatening. Therefore, prompt diagnosis and treatment are warranted to prevent the potential morbidity and mortality of this infectious condition. Emphysematous cystitis is a lower urinary tract infection (UTI) characterized by air within the bladder wall and lumen. 10% of cases of emphysematous cystitis required surgical intervention, including cystectomy or partial cystectomy, whereas the remaining cases were managed with a combination of antibiotics, bladder drainage, and tight glycemic control [1]. The overall death rate of emphysematous cystitis was 7%.

In this report, we present a case of emphysematous cystitis treated with medical management. 

## 2. Case Report 

A 83-year-old woman was introduced to our department with fever elevation and abnormal findings of computed tomography (CT). She was in our hospital due to autoimmune hepatitis, liver cirrhosis, and ascites. She took orally diuretics, antihypertensive, isosorbide mononitrate, and proton pump inhibitor (PPI) as addiction medicine. In addition, prednisolone was administered at an initial dose of  20 mg/day and tapered for the past one month. There was no history of urinary symptoms and UTI. The results of physical examination were unremarkable. 

**Figure 1:**
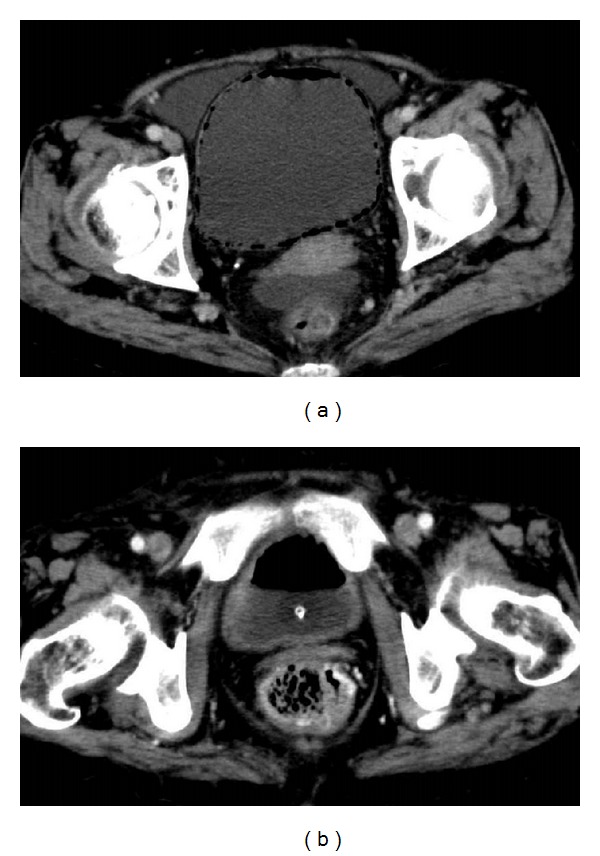
CT findings of the pelvis. (a) CT showed diffuse air within the bladder wall. (b) CT after treatment showed disappearance of air within the bladder wall and normally observed air in the bladder lumen during placement of Foley catheter.

**Figure 2:**
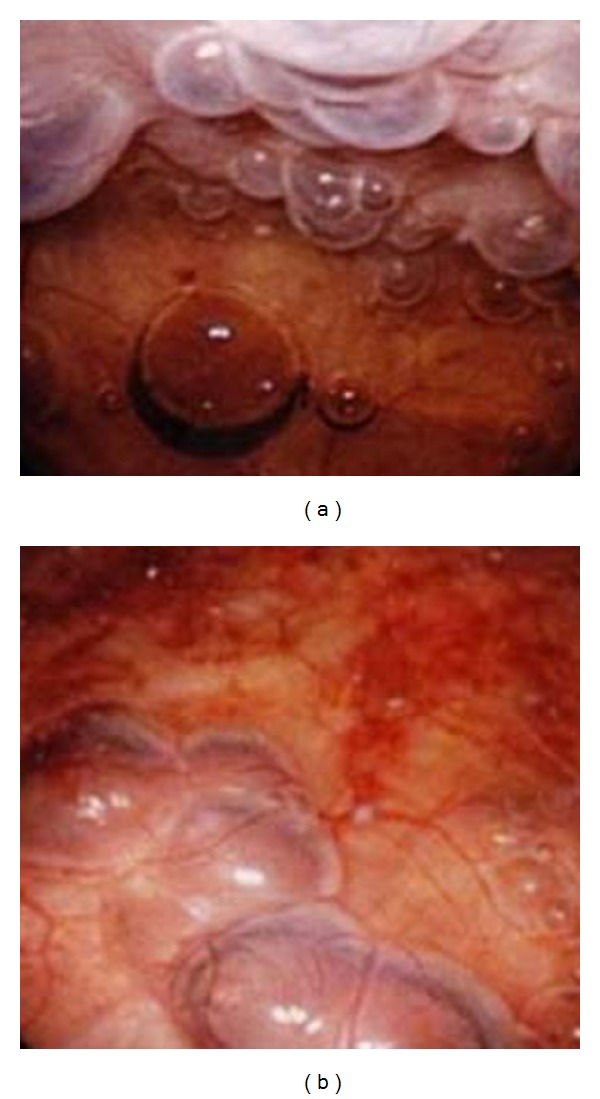
Cystoscopy revealed diffuse submucosal emphysema throughout the bladder.

Pelvic CT revealed diffuse air throughout the bladder wall (Figure 1(a)) and no remarkable findings on bilateral upper urinary tract. Urinalysis showed combined hematuria and pyuria, but no bacteria were identified in urine culture because of prior administration of antibiotics. *Escherichia coli* was detected in blood culture. Additionally, no fungi were isolated from the urine, and the level of beta-D-glucan was within normal limits. In ultrasonography, about 150 mL of residual urine was confirmed. Abnormal findings of complete blood count and laboratory examination included an elevated WBC count (12,200/*μ*L), C-reactive protein (11.7 mg/dL), and creatinine (1.07 mg/dL). On the other hand, fasting blood sugar and hemoglobin A1c were normal level. Cystoscopy confirmed diffuse submucosal emphysema throughout (Figure 2). 

On the basis of diagnosis with emphysematous cystitis, she was treated with antibiotics based on the results of blood culture and indwelling Foley catheter. 

After treatment, the improvement of inflammatory findings and submucosal emphysema on cystoscopy and CT (Figure 1(b)) were achieved. Since residual volume of urine was comparatively high, the transurethral catheter was left. Several months later, she had improved urination. Therefore, we did not make the placement of suprapubic cystostomy. 

## 3. Discussion

Emphysematous cystitis is a lower urinary tract infection characterized by air within the bladder wall and lumen, usually occurring in elderly diabetic females, immunocompromised patients. Thomas et al. [1] identified that two-thirds of all reported cases of emphysematous cystitis until 2006 were diabetic and 64% were women, with a median patient age of  66 years. In the present case, because of impaired liver function and massive ascites caused by liver cirrhosis, high dose of steroid at an initial dose of  20 mg/day was administered and tapered gradually. Regarding the association between the dose of steroids administered and the risk of opportunistic infection, it has been reported that a daily dose of  less than 10 mg (prednisolone equivalent milligrams) or a cumulative dose (prednisolone equivalent milligrams × number of days administered systemic steroids) of  less than 700 mg did not increase the risk of infection [2]. However, more than 10 mg of prednisolone was administered daily, leading to the increase of the risk of UTI according to that previous literature. Furthermore, our case also took PPI orally. A recent study showed that users of PPI had higher rates of UTI than nonusers [3]. Therefore, in the present case, occurrence of emphysematous cystitis might be associated with not only steroids use, but also PPI use.

Clinical features of emphysematous cystitis are nonspecific and vary individually. Some patients are asymptomatic or report only irritative voiding problems or lower abdominal pain, while others present with septic shock. It was reported that 7% of cases in literature were asymptomatic and diagnosed incidentally by abdominal imaging for other concurrent conditions. 

Multiple gas-forming microorganisms can cause emphysematous cystitis. It was reported that various bacterial and fungal organisms were isolated in urine culture with *Escherichia coli* being the most prevalent, followed by *Klebsiella pneumonia* [1, 4, 5]. 

CT of the abdomen can more accurately define the extent and severity of this disease [6]. Abdominal ultrasonography and magnetic resonance imaging are less valuable as imaging modalities because of difficult interpretation. In the present case, CT revealed air within the bladder wall, and cystoscopy confirmed diffuse submucosal emphysema throughout the bladder, suggestive of emphysematous cystitis.

There are several theories on the pathogenesis of these rare gas-forming infections. The combination of the presence of gas-forming organisms, a high tissue glucose concentration, and impaired tissue perfusion all favor the development of emphysematous infections of the urinary tract [7]. It is thought that the high glucose concentration within the tissues acts as a favorable substrate for organisms to produce carbon dioxide through natural fermentation processes [8]. However, this does not account for the significant number of nondiabetic patients with emphysematous infections. In such patients, urinary albumin might act as the substrate for gas production by urinary pathogens [9]. Another theory suggests that an impaired host response, involving vascular compromise and impaired catabolism within the tissues, predisposes patients to gas production within these tissues. Though the pathogenesis is not yet fully understood, a multifactorial etiology of impaired host responses with sugar or protein fermentation seems to be a plausible explanation for the production of gas within the affected tissues. 

The management of emphysematous cystitis depends on the severity of this disease. Most publications of this disorder are single case reports, making a comparison between studies difficult. In a recent analysis of the published literature on emphysematous cystitis in the last 50 years, Thomas et al. [1] found 135 cases for review. The treatment generally consists of antibiotics, bladder drainage, and glycemic control with correction of any underlying comorbid disorders. Broad-spectrum antibiotics are used initially. The precise regimen is then tapered to the sensitivities of the urinary pathogens. Although only 9% of patients were treated successfully with oral antibiotics alone, the most were treated with an initial i.v. antibiotic regimen. 

Patients not responding to medical management or those with severe necrotizing infections might require surgical treatment. None of the patients received surgical treatment alone, whereas 10% had combined surgical and medical management. Surgery involved cystectomy, partial cystectomy, or nephrectomy for combined cases with emphysematous pyelonephritis. Anyway, immediate diagnosis and treatment are necessary because of the rapid progression to bladder necrosis, emphysematous pyelonephritis, urosepsis, and possibly fatal evolution. The overall mortality rate was 7%, which increased to 14% in patients who presented with combined emphysematous cystitis and pyelonephritis. In the present case, the improvement of inflammatory findings and submucosal emphysema on cystoscopy and CT were achieved after conservative treatment without cessation of prednisolone. In conclusion, gas-forming infections of the urinary tract, such as emphysematous cystitis and emphysematous pyelonephritis, are uncommon but potentially life threatening. Therefore, prompt diagnosis and treatment are warranted to prevent the potential morbidity and mortality.
